# Survival comparison between endoscopic and surgical resection for non-ampullary duodenal neuroendocrine tumor (1–2 cm)

**DOI:** 10.1038/s41598-022-19725-0

**Published:** 2022-09-12

**Authors:** Jiebin Xie, Yuan Zhang, Ming He, Xu Liu, Pin Xie, Yueshan Pang

**Affiliations:** 1 Department of Gastrointestinal Surgery, Affiliated Hospital of North Sichuan Medical College, Nanchong, Sichuan People’s Republic of China; 2 Department of Geriatrics, The Second Clinical Medical College of North Sichuan Medical College, Nanchong Central Hospital, Nanchong, 637100 Sichuan People’s Republic of China

**Keywords:** Cancer therapy, Endocrine cancer, Gastrointestinal cancer

## Abstract

The treatment plan for non-ampullary duodenal neuroendocrine tumors (d-NETs) with diameters 1–2 cm remains controversial. We therefore aimed to compare the prognostic effects of endoscopic treatment and surgical resection on non-ampullary d-NETs with 1–2 cm diameters. A total of 373 eligible patients were identified from the Surveillance, Epidemiology, and End Results (SEER) database. Propensity score matching (PSM) was performed to match patients 1:1 according to clinicopathological characteristics. Disease-specific survival (DSS) and overall survival (OS) were calculated. Before PSM, there was no significant difference in DSS or OS (all *P* > 0.05), but the T stage, N stage, and TNM stage were significantly different between the two surgical methods (all *P* < 0.05). After 1:1 PSM, the differences in clinicopathological characteristics were significantly reduced (all *P* > 0.05). Survival analysis showed that tumor grade was correlated with DSS and that age was correlated with OS (all *P* < 0.05); however, the surgical method and other clinicopathological characteristics were not correlated with prognosis (all *P* > 0.05). Subgroup survival analysis of patients with T2N0M0 disease and tumors invading the lamina propria or submucosa showed that the 5-year DSS and OS rates were not significantly different according to the surgical approach (all *P* > 0.05). The surgical approach has no significant effect on the prognosis of patients with non-ampullary d-NETs with 1–2 cm diameters, especially those with T2N0M0 disease. This suggests that endoscopic treatment may be a preferred option for these patients.

## Introduction

Duodenal neuroendocrine tumors (d-NETs), tumors originating from neuroendocrine cells, account for approximately 3% of all gastrointestinal neuroendocrine tumors. The incidence of d-NETs has increased significantly in recent years with the popularization of endoscopy. World Health Organization (WHO) classification, lymph node status and tumor size have historically been important prognostic factors^1–4^. A recent study^1^ reported that the incidence of lymph node metastasis (LNM) increased with d-NETs size (≤ 1 cm: 40.0% vs 1–2 cm: 65.7% vs > 2 cm: 80.0%). Therefore, currently, the choice of surgical regimen is based mainly on tumor size, depth of invasion, primary site, and the presence of lymph node metastasis (LNM) on imaging^5,6^. For tumors 1 cm or less in diameter that are not in the periampullary region and in which LNM is not suspected, endoscopic treatment is recommended. For tumors larger than 2 cm in diameter, surgical resection is recommended. For tumors in the periampullary region, whose biological behaviors differ from those of neuroendocrine tumors (NETs) in other regions, endoscopic treatment and lymph node biopsy or surgical resection are required. For tumors with diameters greater than 1–2 cm, endoscopic treatment has the significant advantages of minimal invasiveness, low cost, short hospital stay, and less impact on quality of life^7,8^; however, it also has the risks of bleeding, perforation, positive margins, and missed metastatic lymph nodes^7,9–11^. Although surgical resection can more completely remove the primary lesion and LNM, while its cost is high and postoperative complications are more common^2,12,13^. Due to the lack of prospective studies and large clinical trials, the specific treatment plan is still not standardized^5^, and their prognosis are still rarely reported. Therefore, further studies with large sample sizes are needed to confirm whether endoscopic treatment or surgical resection is best.

The Surveillance, Epidemiology, and End Results (SEER) database is a tumor registry database that was established in the 1970s in the United States and covers approximately 27.8% of cancer patients. The database provides important data support for clinical research and clinical decision-making. However, since the SEER database includes data from multiple cancer registration centers, the real-world noncontrolled data are imbalanced to a certain extent, and much information is missing. To balance the baseline characteristics between groups, this study collected the clinicopathological and prognostic data of non-ampullary d-NET patients in the SEER database with tumors 1–2 cm in diameter. The propensity score matching (PSM) method was used to match the characteristics between the groups to investigate whether surgical resection improved the long-term prognosis of patients.

## Materials and methods

### Ethics statement

The study was complied with the Declaration of Helsinki. Because all the data were derived from a public database and individual information was anonymous, and it has been permitted to obtain the data from the SEER database (Reference Number 13907-Nov2020), the ethical committee waved away the formal ethical approval.

### Patient selection and data collection

There are three SEER registry systems, SEER 9, SEER 13 and SEER 18, and include patient demographics, tumor characteristics (histology, grade, tumor-node-metastasis stage), treatment and patient vital statuses, covering approximately 9.4%, 13.4% and 27.8% of the American population, respectively. This database is available for public cancer studies.

The data on patients who were pathologically diagnosed with non-ampullary d-NETs (International Classification of Diseases for Oncology, third edition (ICD-O-3), Primary Site-labeled: C17.0-Duodenum) between 2004 and 2018 were collected from the SEER 18 Researcher database by using SEER*Stat 8.3.9 software (https://seer.cancer.gov/seerstat/) in our study, which was submitted in April 2021 (ICD-O-3 histology code, 8240: Carcinoid tumor, 8153: Gastrinoma, 8249: Atypical Carcinoid tumor). All patients with a tumor 1–2 cm in diameter who underwent endoscopic treatment (local tumor destruction: RX Summ-Surg Prim Site (1998+) codes 11: photodynamic therapy, 12: electrocautery; fulguration, 13: cryosurgery, 14: laser; and local tumor excision codes 21: photodynamic therapy (PDT), 22: electrocautery, 23: cryosurgery, 24: laser ablation, 25: laser excision, 26: polypectomy, 27: excisional biopsy) or surgical resection with or without lymphadenectomy (code 30: simple/partial surgical removal of primary site, 40: total surgical removal of primary site and 60: partial or total removal of the primary site with an en bloc resection (partial or total removal) of other organs) were enrolled. The exclusion criteria were as follows: (1) tumor size was 1 cm or less than 1 cm or greater than 2 cm; (2) unknown T stage, N stage or have metastatic disease; (3) unclear cause of death; and (4) no surgical resection or a lack of surgical details. Due to the strict register-based nature of the study, the requirement for informed consent was waived.

Demographic characteristics, such as sex, age, and race, and pathological characteristics, such as tumor grade, T stage, N stage, M stage, tumor size, CS extension, and histologic type ICD-O-3, were collected. According to detailed information such as tumor size and CS extension provided by the SEER database, the T stage of patients was rejudged according to the eighth edition of the American Joint Committee on Cancer (AJCC) stage classification system. All data collection and statistical calculation procedures were independently completed by two authors (Jiebin Xie and Yueshan Pang). Disease-specific survival (DSS) and overall survival (OS) were also calculated from the date of surgery.

### Statistical analysis

Continuous and categorical variables are expressed as the mean ± SD and total (percentage), respectively. The chi-squared test, Fisher’s exact test or the t-test was used to quantify the differences between surgical groups. The Kaplan–Meier method with the log-rank test was used for survival analysis. Statistical analysis was performed using SPSS 22.0 (Chicago, IL, USA). *P* < 0.05 was considered statistically significant (two-tailed tests). The MatchIt package of R software v3.6.3 (https://www.R-project.org) was used to perform 1:1 PSM with a caliper value set to 0.1 for sex, race, income, tumor size, T stage, N stage, M stage, TNM stage and histologic type^14^. The nearest-neighbor matching method was used to match the baseline characteristics between the two groups.

## Results

### General condition and survival analysis before matching

**Figure 1:**
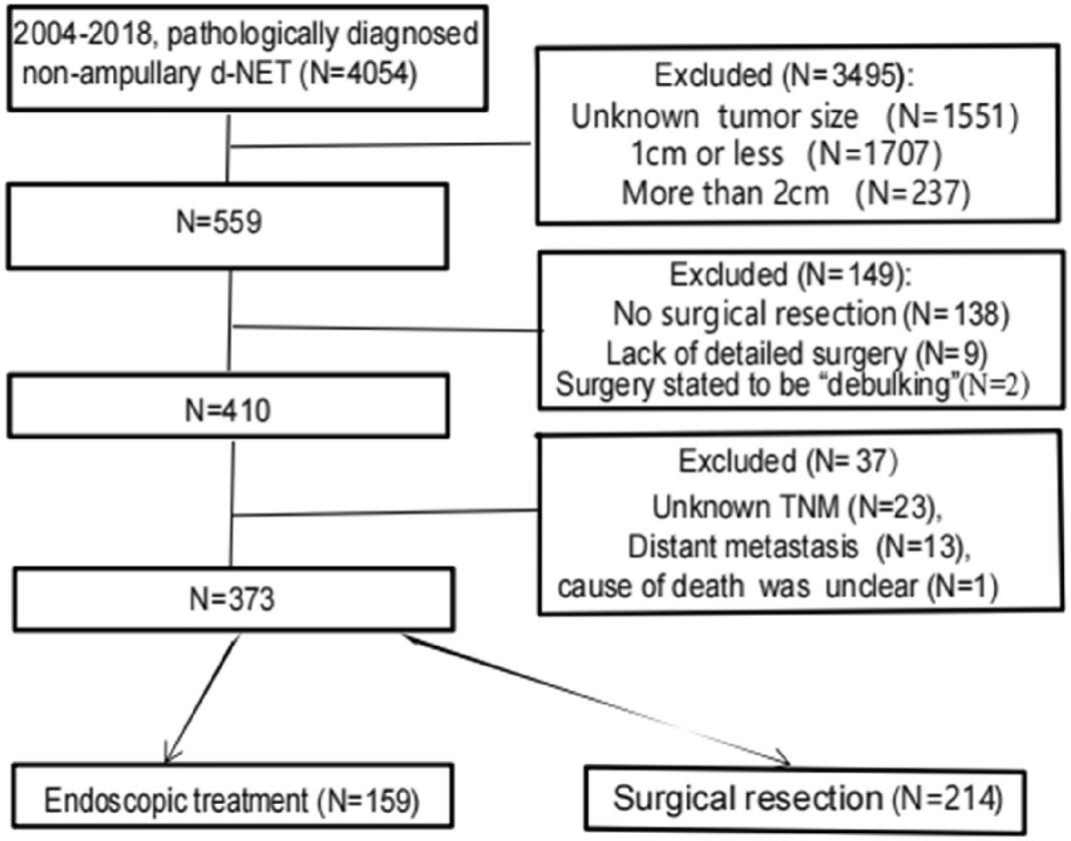
Flow chart of patient cohort selection.

**Figure 2:**
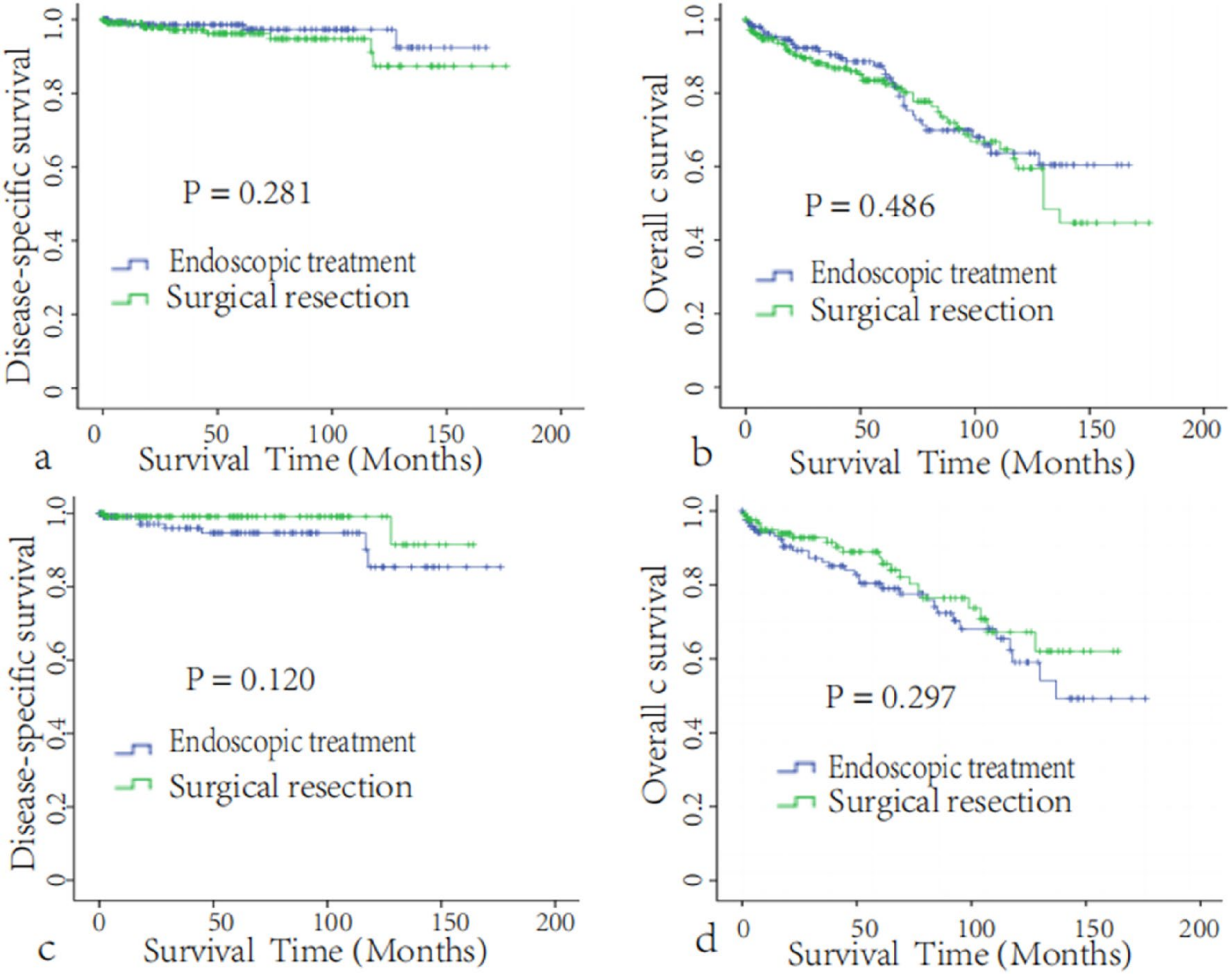
Disease-specific survival (**a**) and overall survival (**b**) of the endoscopic treatment group and surgical resection group before PSM (n = 373); disease-specific survival (**c**) and overall survival (**d**) of the endoscopic treatment group and surgical resection group after PSM (n = 248).

Data on 4045 patients who were pathologically diagnosed with non-ampullary d-NETs between 2004 and 2018 were obtained from the SEER 18 database. After patients were screened by the above inclusion and exclusion criteria, 373 d-NET patients met the inclusion criteria: 159 underwent endoscopic treatment, and 214 underwent surgical resection (Fig. 1), including 156 simple/partial surgical removal of primary site (65.1%, code 30), 38 radical surgery (22.6%, code 60), 20 total surgical removal of primary site (11.3%, code 40). Among all tumors, 89.8% were T2, 20.6% were N1 (Table 1). This study cutoff was on December 31, 2019, The median follow-up time was 51 months, and the overall 5-year DSS and OS rates were 97.3 ± 1.0% and 84.7 ± 2.2%, respectively. The 5-year DSS and OS rates of patients in the endoscopic treatment group were 98.6 ± 1.0% and 86.4 ± 3.1%, respectively. The 5-year DSS and OS rates of patients in the surgical resection group were 96.2 ± 1.6% and 83.4 ± 2.9%, respectively. Kaplan–Meier survival analysis showed no significant difference in the survival rate between the two groups before PSM (all *P* > 0.05, Fig. 2a,b).

**Table 1:** Distribution profiles of the clinicopathologic factors of the patients in the endoscopic treatment group and surgical resection group before and after PSM.

Characteristics	Cases (%)	Before PSM			After PSM		
		ET (n = 159)	SR(n = 214)	*P*-value	ET (n = 124)	SR(n = 124)	*P*-value
Age, (year x ± s)	62.7 ± 12.3	63.7 ± 12.4	62.0 ± 12.3	0.188	63.6 ± 11.5	62.3 ± 13.2	0.558
Size, (mm x ± s)	14.8 ± 2.9	14.9 ± 3.0	14.8 ± 2.8	0.549	14.8 ± 2.8	14.6 ± 2.8	1
**Sex**				0.285			1
Male	204 (54.7)	92 (62.0)	112 (52.8)		74 (59.7)	74 (59.7)	
Female	169 (45.3)	67 (38.0)	102 (47.2)		50 (39.3)	50 (39.3)	
**Race**				0.533			0.595
White	250 (67.0)	103 (61.1)	147 (69.2)		84 (67.7)	83(66.9)	
Black	92 (24.7)	40 (26.9)	52 (25.1)		33 (26.6)	30 (24.2)	
Others	31 (8.3)	16 (12.0)	15 (5.7)		7 (5.6)	11 (8.9)	
**T stage**				0.001			0.845
T2	335 (89.8)	156 (97.2)	179 (82.1)		122 (96.8)	121 (94.7)	
T3	30 (8.0)	2 (1.9)	28 (14.3)		1 (2.1)	2 (5.3)	
T4	8 (2.1)	1 (0.9)	7 (3.6)		1 (1.1)	1 (0)	
**N stage**				< 0.001			0.783
N0	296(79.4)	153(93.5)	143 (58.5)		116 (93.5)	118 (95.2)	
N1	77 (20.6)	6 (6.5)	71 (41.5)		8 (6.5)	6 (4.8)	
**TNM stage**				< 0.001			0.796
II	292 (78.3)	152 (91.6)	140 (56.4)		115 (92.7)	117 (94.4)	
III	81 (271.7)	7 (6.5)	74 (38.5)		9 (7.3)	7 (5.6)	
**Grade**				0.06			0.128
Grade I	235(63.0)	91 (88.9)	144 (79.5)		80 (64.5)	77 (62.1)	
Grade II	30 (8.0)	8 (10.2)	22 (17.9)		7 (5.6)	16 (12.9)	
Grade III	3 (0.8)	1 (0.9)	2 (2.6)		0 (0)	0 (0)	
Unknown	105 (28.2)	59	46		37 (29.8)	31 (25.0)	
**Histologic type**^**a**^				0.856			0.601
8240	356 (95.4)	152 (72.2)	204 (70.3)		120 (96.8)	118 (95.2)	
8153	11 (2.9)	4 (3.7)	7 (3.6)		3 (2.4)	3 (2.4)	
8249	6 (1.6)	3 (1.9)	3 (0.5)		1 (0.8)	3 (2.4)	
**Income**				0.130			0.254
< $50,000	87 (23.3)	40 (25.2)	47 (22.0)		26 (21.0)	28 (22.6)	
$50,000–74,999	184 (49.3)	69 (43.4)	115 (53.7)		71 (57.3)	59 (47.6)	
$75,000+	102 (27.3)	50 (31.4)	52 (2)		27 (21.8)	37 (29.8)	
30-day mortality	10 (2.7)	4 (2.5)	6 (2.8)	0.865	3 (2.4)	4 (3.2)	0.701
90-day mortality	24 (6.4)	11 (6.9)	13 (6.1)	0.743	9 (7.2)	11 (8.9)	0.641
5-year CSS	97.3 ± 1.0	98.6 ± 1.0	96.2 ± 1.6	0.281	96.5 ± 2.7	98.9 ± 1.1	0.120
5-year OS	84.7 ± 2.2	86.4 ± 3.1	83.4 ± 2.9	0.486	82.4 ± 5.0	88.5 ± 3.7	0.297

*ET* endoscopic treatment, *SR* surgical resection, *PSM* propensity score matching, *WHO* World Health Organization, *Grade I* well differentiated, *Grade II* moderately differentiated, *Grade III* poorly differentiated or undifferentiated.

^a^International Classification of Diseases for Oncology, 3rd Edition (ICD-O-3): 8240, Carcinoid tumor; 8153, Gastrinoma, 8249, Atypical Carcinoid tumor.

### Comparison of baseline data and prognosis of the endoscopic treatment and surgical resection groups before and after matching

Before matching, there was no significant difference in the mean age between the endoscopic treatment group (63.7 ± 12.4 years) and the surgical resection group (62.0 ± 12.3 years, *P* = 0.188). The mean tumor sizes were similar (endoscopic treatment group 14.9 ± 3.0 mm and surgical resection group 14.8 ± 2.8 mm, *P* = 0.549). The T stage, N stage, and TNM stage in the surgical resection group were significantly higher than those in the endoscopic treatment group (all *P* < 0.001). The differences in race, sex, tissue type, 30-d mortality, 90-day mortality, tumor grade and income were not statistically significant between the two groups. To eliminate the differences in baseline characteristics between the two groups, PSM was used to balance sex, race, tumor size, T stage, N stage, income, TNM stage, tumor grade and histologic type. A total of 248 patients were selected according to the chosen 1:1 ratio, with 124 in each group. On this basis, the comparison of patients in the matched groups showed that the differences in clinicopathological characteristics were significantly reduced, and none of the above characteristics were significantly different between the two groups after matching (Table 1).

### Survival analysis after matching

**Figure 3:**
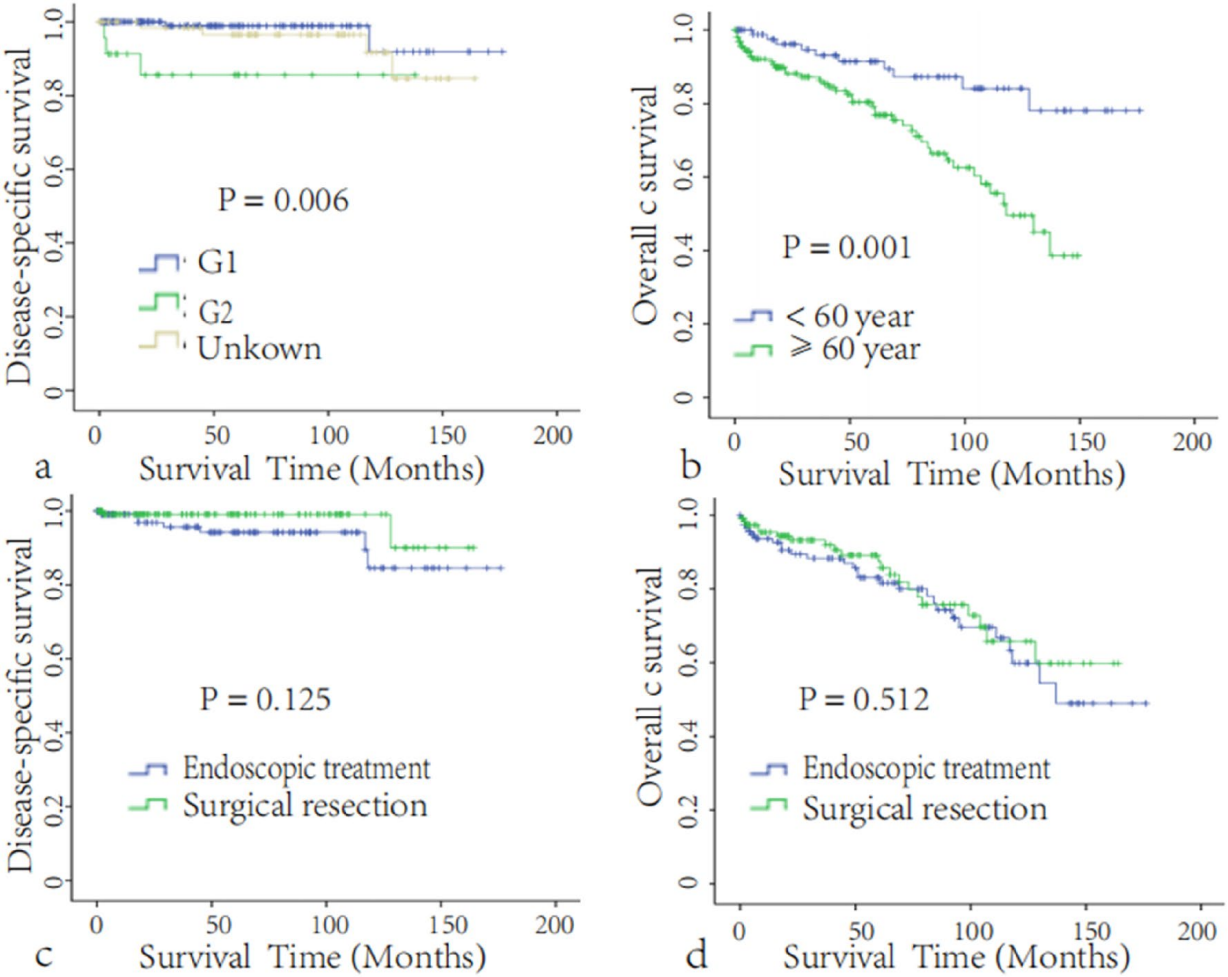
(**a**) Disease-specific survival according to tumor grade after PSM (n = 248). (**b**) Overall survival according to age after PSM (n = 248). Subgroup survival analysis of patients with T2N0M0 disease. Disease-specific survival (**c**) and overall survival (**d**) of the endoscopic treatment group and surgical resection group after PSM (n = 231).

After matching, the 5-year DSS rates of patients in the endoscopic treatment and surgical resection groups were 96.5 ± 2.7% and 98.9 ± 1.1%, respectively. Survival analysis also showed no significant difference in DSS or OS between the two groups (*P* = 0.120, 0.297, Fig. 2c,d). The tumor grade was correlated with DSS (*P* = 0.006, Fig. 3a), and age was correlated with OS (*P* = 0.001, Fig. 3b), but the surgical approach and other clinicopathological features, such as race, sex, income, TNM stage, and tissue type, were not correlated with DSS or OS (Table 2).

**Table 2:** Factors associated with disease-specific survival and overall survival after PSM for non-ampullary duodenal neuroendocrine tumors (d-NETs).

Characteristics	Cases (%)	DSS			OS		
	Total (n = 248)	5-year DSS (%)	χ^2^	P-value	5-year OS (%)	χ^2^	P-value
**Age (year)**			0.289	0.591		12.09	0.001
< 60	88 (35.5)	96.9 ± 2.2			91.4 ± 3.4		
≥ 60	160 (64.5)	96.9 ± 1.6			79.2 ± 3.7		
**Sex**			0.894	0.345		0.031	0.859
Male	148 (59.7)	95.7 ± 1.9			84.8 ± 3.3		
Female	100 (40.3)	98.5 ± 1.5			81.8 ± 4.7		
**Size (cm)**			0.019	0.892		0.606	0.136
≤ 1.5	177 (71.4)	97.2 ± 1.4			81.0 ± 3.5		
> 1.5	71 (21.6)	96.2 ± 2.7			89.8 ± 4.0		
**Race**			1.777	0.411		2.283	0.319
White	167(67.3)	96.0 ± 1.8			84.9 ± 3.1		
Black	63 (25.4)	98.3 ± 1.7			80.1 ± 6.1		
Others	17 (7.3)	100			86.3 ± 9.2		
**Grade**			10.227	0.006		1.972	0.373
Grade I	157(63.3)	98.9 ± 1.0			86.1 ± 3.5		
Grade II	23(9.3)	85.6 ± 7.8			77.0 ± 9.1		
Unkown	68 (27.4)	96.5 ± 2.4			83.1 ± 4.6		
**Histologic type**^**a**^			0.171	0.982		0.99	0.804
8240	240 (96.8)	96.7 ± 1.4			82.9 ± 2.8		
8249	3 (1.2)	100			50.0 ± 35.4		
8153	5 (2.0)	100					
**Surgical procedure**			2.411	0.120		1.086	0.297
Surgical resection	124 (50.0)	99.1 ± 0.9			87.3 ± 3.5		
Endoscopic treatment	124 (50.0)	94.7 ± 2.4			80.4 ± 4.0		
**TNM stage**			0.7	0.403		0.548	0.459
II	232 (93.5)	96.6 ± 1.4			83.8 ± 2.8		
III	16 (6.5)	100			60.2 ± 14.1		
**Income**			1.624	0.444		0.099	0.952
< $50,000	54 (21.8)	98.9 ± 1.9			87.1 ± 5.1		
$50,000–74,999	130 (52.4)	95.6 ± 2.2			83.0 ± 3.8		
$75,000+	64 (25.8)	98.4 ± 1.6			81.9 ± 5.7		

*DSS* disease-specific survival, *OS* overall survival, *PSM* propensity score matching.

^a^International Classification of Diseases for Oncology, 3rd Edition (ICD-O-3): 8240, Carcinoid tumor; 8153,Gastrinoma, 8249, Atypical Carcinoid tumor. WHO: World Health Organization. Grade I,well differentiated; Grade II, moderately differentiated.

### Subgroup survival analysis

Approximately 78.8% (279/373) of d-NET patients with tumor diameters between 1 and 2 cm had T2N0M0 disease, which can be potentially treated by endoscopic treatment. Therefore, further subgroup survival analysis was also performed in T2N0M0 patients after matching. In line with previous results, the 5-year DSS and OS (Fig. 3c,d) rates were not significantly different according to the surgical approach, sex, race, income, tumor size or histology type; however, the tumor grade was still correlated with DSS, and age was correlated with OS (Table 3).

**Table 3:** Factors associated with disease-specific survival and overall survival after PSM for non-ampullary duodenal neuroendocrine tumors (d-NETs) patients with T2N0M0 stage.

Characteristics	Cases (%)	DSS			OS		
	Total (n = 231)	5-year DSS	χ^2^	P-value	5-year OS	χ^2^	P-value
**Age (year)**			0.126	0.723		10.653	0.001
< 60	58 (34.3)	96.5 ± 2.5			93.7 ± 3.1		
≥ 60	111 (65.9)	96.8 ± 1.8			80.8 ± 3.7		
**Sex**			0.854	0.355		0.167	0.683
Male	100(59.2)	95.4 ± 2.0			85.6 ± 3.3		
Female	69 (40.8)	98.4 ± 1.6			84.5 ± 4.6		
**Size (cm)**			0.068	0.795		0.21	0.647
≤ 1.5	118 (69.8)	97.0 ± 21.5			82.8 ± 3.4		
> 1.5	51 (30.2)	95.8 ± 2.9			90.9 ± 3.9		
**Race**			1.799	0.407		2.604	0.272
White	114 (67.5)	95.6 ± 1.9			85.9 ± 3.1		
Black	46 (27.2)	98.3 ± 1.7			82.1 ± 5.9		
Others	9 (5.3)	100			93.3 ± 6.4		
**Grade**			9.094	0.011		2.243	0.326
Grade I	144(85.2)	98.8 ± 1.2			88.5 ± 3.4		
Grade II	24(14.2)	85.6 ± 7.8			77.0 ± 9.1		
Unknown		96.4 ± 2.5			82.5 ± 4.8		
**Histologic type**^**a**^			0.169	0.919		0.411	0.814
8240	132 (78.1)	96.5 ± 1.4			84.8 ± 2.8		
8153	35 (20.7)	100			100		
8249	2(1.2)	100			50.0 ± 35.4		
**Surgical procedure**			2.354	0.125		0.429	0.512
Surgical resection	84 (49.7)	99.1 ± 0.9			87.4 ± 3.7		
Endoscopic treatment	85 (50.3)	94.2 ± 2.5			83.1 ± 3.9		
**Income**			1.729	0.421		0.107	0.948
< $50,000	54 (21.8)	97.9 ± 2.1			88.4 ± 5.1		
$50,000–74,999	130 (52.4)	95.2 ± 2.4			83.6 ± 3.9		
$75,000+	64 (25.8)	98.3 ± 1.7			85.6 ± 5.3		

*DSS* disease-specific survival, *OS* overall survival, *PSM* propensity score matching.

^a^International Classification of Diseases for Oncology, 3rd Edition (ICD-O-3): 8240, Carcinoid tumor; 8153,Gastrinoma, 8249, Atypical Carcinoid tumor. WHO: World Health Organization. Grade I, well differentiated; Grade II, moderately differentiated.

**Figure 4:**
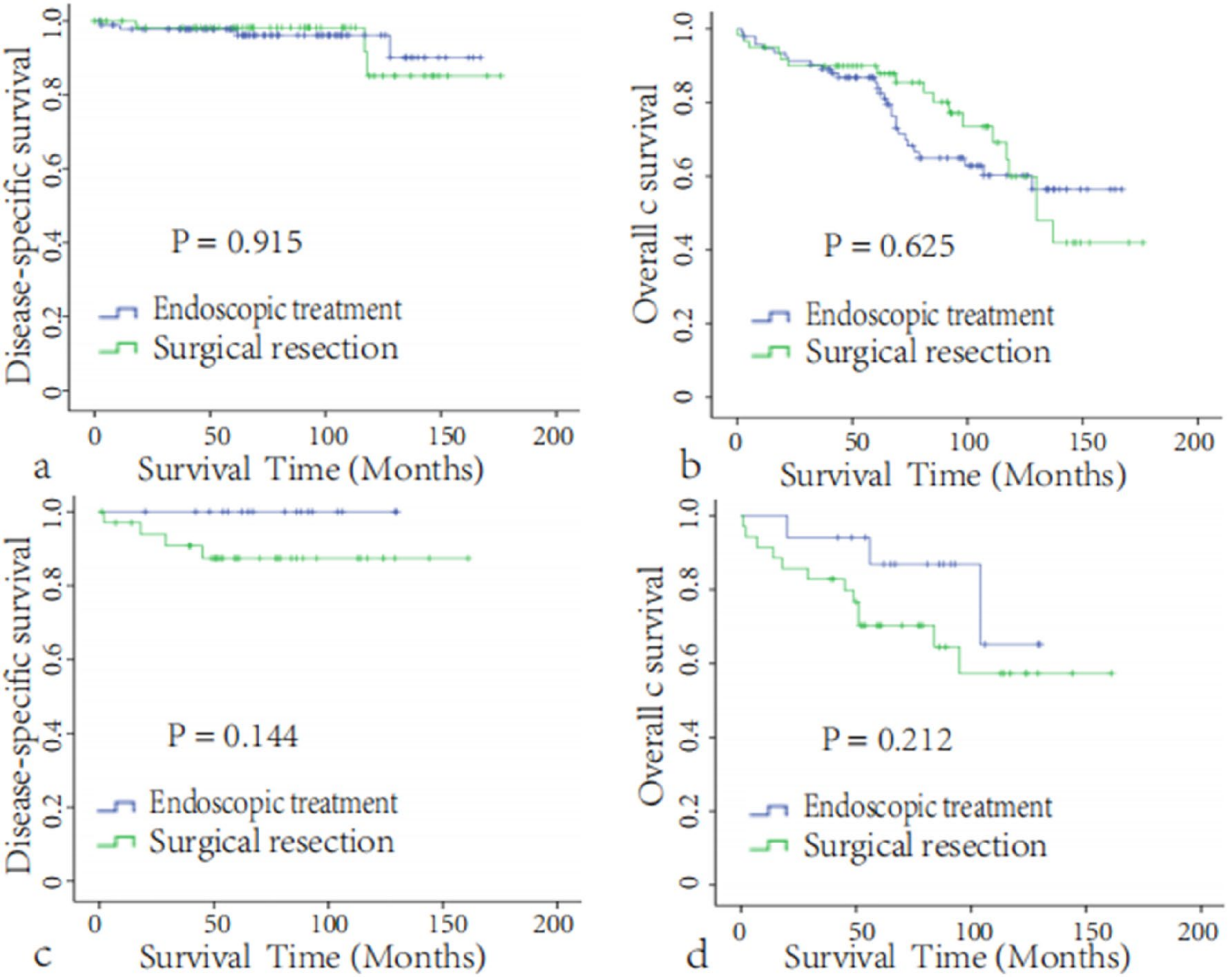
Disease-specific survival (**a**) and overall survival (**b**) of the endoscopic treatment group and surgical resection group for the tumor limiting to the mucosa or submucosa (n = 153). Disease-specific survival (**c**) and overall survival (**d**) of the endoscopic treatment group and surgical resection group for the tumorinvading into the muscularis propria (n = 52).

Then, the patients during 2004–2015 (SEER database did not provide the data of tumor invasion after 2016) with T2N0M0 stage were further divided into two subgroups according to the depth of tumor invasion: group one in which the tumor was limited to the mucosa or submucosa; group two in which the tumor was invaded the muscularis propria to further investigate the validity of endoscopic resection for T2N0M0 lesions. Except for tumor grade in group one, there were no significant differences in age, tumor size, sex, histologic type, income, 30-d mortality or 90-day mortality between the endoscopic treatment group and the surgical resection group.We found that endoscopic resection was used more frequently for lesions with invasion limited to the mucosa or submucosa and surgical resection was used more frequently for lesions invaded the muscularis propria (Table 4); however, regardless of the depth of tumor invasion, as expected, the 5-year DSS or OS (Fig. 4) rates were not significantly different according to the surgical approach, in keeping with previous results.

**Table 4:** Distribution profiles of the clinicopathologic factors of the patients in the endoscopic treatment group and surgical resection group in different depth of tumor invasion.

Characteristics	Non-MP (N = 153)			MP (N = 52)		
	SR (N = 60)	ET(N = 93)	*P*-value	SR (N = 35)	ET (N = 17)	*P*-value
**Age (year)**			0.573			0.628
< 60	20 (33.3)	27 (29.0)		12 (34.3)	7 (41.2)	
≥ 60	40 (66.7)	63 (71.0)		23 (65.7)	10 (58.8)	
**Sex**			0.566			0.476
Male	23 (38.3)	43 (43.0)		16 (45.7)	6 (35.3)	
Female	37 (61.7)	53 (57.0)		19 (54.3)	11 (64.7)	
**Size (cm)**			0.607			0.78
≤ 1.5	43 (71.7)	63 (67.7)		24 (68.6)	11 (64.7)	
> 1.5	17 (28.3)	30 (32.3)		11 (31.4)	6 (35.3)	
**Race**			0.073			0.381
White	44 (73.3)	60 (64.5)		25 (71.4)	9 (52.9)	
Black	15 (25.0)	22 (23.7)		8 (22.9)	7 (41.2)	
Others	1 (1.7)	11 (13.8)		2 (5.7)	1 (5.9)	
**Tumor grade**			0.036			0.088
Grade I	37 (61.7)	40 (43.0)		18 (51.4)	6 (35.3)	
Grade II	6 (10.0)	5 (5.4)		7 (20.0)	1 (5.9)	
Grade III	0 (0)	1 (1.1)				
Unknown	17 (28.3)	47 (50.5)		10 (28.6)	10 (58.8)	
**Histologic type**^**a**^			0.956			1
8240	58 (96.7)	89 (95.7)		35 (100.0)	17 (100)	
8153	1 (1.7)	2 (2.2)		0 (0)	–	
8249	1 (1.7)	2 (2.2)		0 (0)	0 (0)	
**Income**			0.167			0.115
< $50,000	21 (35.0)	26 (28.0)		7 (20.0)	4 (23.5)	
$50,000–74,999	29 (48.3)	39 (41.9)		22 (62.9)	6 (35.3)	
$75,000+	10 (16.7)	28 (30.1)		6 (17.1)	7 (41.2)	
30-day mortality	1 (1.7)	0 (0)	0.212	0 (0)	0 (0)	1
90-day mortality	2 (3.3)	3 (3.2)	0.971	2 (5.7)	0 (0)	0.315
5-year CSS	98.2 ± 1.8	97.8 ± 1.5	0.915	87.4 ± 5.9	100	0.144
5-year OS	89.9 ± 3.9	85.3 ± 3.8	0.625	70.3 ± 7.9	86.9 ± 8.7	0.212

*ET* endoscopic treatment, *SR* surgical resection, *PSM* propensity score matching, *WHO* World Health Organization, *Grade I* well differentiated, *Grade II* moderately differentiated, *Grade III* poorly differentiated or undifferentiated.

^a^International Classification of Diseases for Oncology, 3rd Edition (ICD-O-3): 8240, Carcinoid tumor; 8153,Gastrinoma,8249, Atypical Carcinoid tumor. MP, tumor invading into the muscularis propria (MP); non-MP, tumor limiting to the mucosa or submucosa.

## Discussion

In the present study, we used the SEER database to determine DSS and OS and mortality outcomes in patients with diameters between 1 and 2 cm non-ampullary d-NETs who underwent endoscopic versus surgical resection. This is one of the largest studies to date. No matter before or after PSM, we found that patients who underwent surgical resection compared with endoscopic excision had no survival benefit, and there were no significant differences in 30-day and 90-day mortality between the two surgical approaches. Only tumor grade was correlated with DSS and age was correlated with OS. Even if the tumor invades the muscularis propria, endoscopic treatment is not inferior to surgical resection in terms of long-term survival for T2N0M0 lesions.

Patients with tumor diameters of 1–2 cm have a higher likelihood of lymphatic metastasis^1,4^ and it is challenging for endoscopists to deal with the d-NETs because they have a thinner wall in the duodenum than in other gastrointestinal tracts^10^. Therefore, the rate of surgical resection is currently higher for these patients as our results (214/159). However, there is no consensus for the treatment of d-NETs 1–2 cm in diameter^15^, the differences in the effects of endoscopic vs. surgical treatment on prognosis have been rarely reported. Margonis et al.^2^ studied 146 d-NET patients undergoing different surgical approaches and found that the prognosis of d-NET was correlated with tumor grade and metastasis at the time of diagnosis but not with the surgical approach. Our study showed that among the 373 patients who met the inclusion criteria, there was also no significant difference in DSS or OS between the two groups before matching. However, fewer of our patients underwent endoscopic treatment than surgical resection, and endoscopic treatment was performed mainly in well-differentiated, older patients and those with T2 stage and N0 tumors. Considering the differences in the clinicopathological characteristics, sample size, and risk factors before treatment between patients in the endoscopic treatment group and surgical resection group, which made it difficult to balance the covariates between the groups, PSM was used to control the resulting bias^2^. PSM can simultaneously be used to match multiple characteristics, minimize confounding bias, and better simulate clinical studies, especially when we are unable to perform a prospective clinical study or if the clinical study is of low quality. An analysis based on a large sample size after PSM has more reference value^16^. In the present study, after PSM, there was no significant difference in the clinicopathological characteristics between the two groups, which improved the reliability of the conclusions of the subsequent analyses.

After matching, our results showed that the surgical approach, tumor size, sex, race, income, TNM stage, and tissue type were not correlated with either DSS or OS, but the tumor grade was correlated with prognosis (*P* < 0.006), in line with the findings reported by Margonis et al.^14^, and patients aged less than 60 years had better 5-year OS than those aged older than 60 years (*P* = 0.001). In view of surgical resection is too aggressive and accompanied by serious surgical risks for the local disease, endoscopic treatment, as the main treatment method for tumors ≤ 1 cm, has the significant advantages of a short operation time, low cost, short hospital stay, and low impact on quality of life^7,8^. With advancements in preoperative staging technology such as ultrasound endoscopy, multiphasic CT, and 68Ga-DOTA-SSA-PET-CT^17^, the advantages of endoscopic treatment (endoscopic mucosal resection, endoscopic submucosal dissection and endoscopic full-thickness resection) of gastrointestinal NET tumors have become obvious^18^. Especially in recent years, with the application of endoscopic full-thickness resection technology, the potential curative treatment of lesions involving any layer of the duodenal wall has gradually increased^19–24^. The study of Dwyer S^24^ showed that endoscopic full-thickness resection can be used for well-differentiated T2 neuroendocrine tumors in the duodenal bulb lesions ≥ 2.0 cm, and no evidence of disease recurrence occurred during the follow-up.

However, due to the thin wall of the duodenum and its rich blood vessels, most tumors invade the submucosa. Therefore, there are risks of bleeding, perforation, positive margins, and missed metastatic lymph nodes in endoscopic treatment^10,11,25^. And some studies reported that the lymphatic metastasis rate of d-NETs with a diameter of 1–2 cm was approximately 60%^1,26^. Although, our data showed that the positive rate was only 20.6% (including 33.1% of the surgical resection group and 3.7% of the endoscopic treatment group. This difference may come from earlier studies’ patients all underwent surgical resection). These data still suggest that d-NETs with diameters of 1–2 cm have a higher rate of LNM, therefore, using appropriate imaging methods to exclude local periduodenal lymph node metastases is very important before endoscopic treatment.

To further analyze the survival advantage of endoscopic treatment in patients with T2N0M0 d-NETs, patients with LNM or T2+ were excluded from the stratified analysis, and the results still showed no difference in survival between patients who underwent surgical resection and those who underwent endoscopic treatment. Surgical resection has no survival benefit over endoscopic treatment, except that tumor grade and age are related to prognosis. However, for fear of positive margins, endoscopic treatment is mainly used for lesions limited to mucosal or submucosal lesions, and surgical resection is currently used more frequently for lesions with muscularis propria invasion as our results showed that approximately 2/3(35/52) patients were treated by surgical resection. To investigate the validity of endoscopic resection for T2N0M0 lesions, these patients were further stratified into two subgroups according to the depth of invasion, Kaplan–Meier survival curves showed for the first time that the long-term efficacy of endoscopic treatment was no less than that of surgical resection regardless of the depth of tumor invasion. At the same time, Gincul Rodica et al.^27^ reported that only two of nine R1 d-NETs patients without additional surgery developed recurrence during the 56-month (range 6–175 months) follow-up period after endoscopic treatment. Therefore, our results suggest that endoscopic treatment is the preferred option for T2N0M0 patients with well-differentiated and a diameter of 1–2 cm. However, duodenal endoscopic submucosal dissection or endoscopic full-thickness resection is truly challenging, and even Japanese experts think twice before it is indicated^28^, Our results should be treated with caution in clinical practice, especially for patients with tumor invading muscularis.

This study has some limitations. First, it was a retrospective study based on the SEER database, and thus residual confounding cannot be excluded. Second, the SEER database does not provide information on the type of work-up (echoendoscopy, MRI, CT scan…) performed before the surgery or endoscopic treatment, or the type of endoscopic procedure (EMR or endoscopic submucosal dissection or endoscopic full-thickness resection). Some important prognostic factors such as the Ki67 index, lymphovascular invasion, mitotic count, R0/R1 status were also not available in the SEER database. Third, to ensure the integrity of the data, we excluded many patients, so the total sample size was small. In particular, the numbers of patients with N1, T3, and T4 disease after matching were small. Finally, the advantages and disadvantages of the two surgical methods are important factors in their short-term efficacy, but the SEER database does not provide information on postoperative complications, which limits the comparison of short-term efficacy. Although our data are not ideal, PSM was able to well balance the clinical and pathological characteristics of the two groups, reducing selection bias. We also compared the differences in OS between the two groups and achieved consistent results with previous studies. Our results still need to be validated in a prospective, multicenter, randomized controlled study.

## Conclusion

The surgical approach had no significant effect on prognosis, but age and the tumor grade were independent prognostic factors in non-ampullary d-NET patients with a maximum tumor diameter of 1–2 cm. This suggests that endoscopic treatment may be a preferred option for T2N0M0 disease with G1.

## Data Availability

The datasets analyzed in the present study can be obtained from the Surveillance, Epidemiology, and End Results (SEER) program online website (https://seer.cancer.gov/). The datasets are also available from the corresponding author upon reasonable request.
